# Zr-Based Biocomposite Materials as an Alternative for Fluoride Removal, Preparation and Characteristics

**DOI:** 10.3390/polym14081575

**Published:** 2022-04-12

**Authors:** Adriana Robledo-Peralta, Linda Viviana García-Quiñonez, René I. Rodríguez-Beltrán, Liliana Reynoso-Cuevas

**Affiliations:** 1 Department of Sustainable Engineering, Advanced Materials Research Center (CIMAV-Durango), CIMAV 110 Street, Ejido Arroyo Seco, Durango C.P. 34147, Durango, Mexico; adriana.robledo@cimav.edu.mx; 2 CONACYT-Centro de Investigación Científica y de Educación Superior de Ensenada, Unidad Foránea Monterrey, Alianza Centro 504, PIIT, Apodaca C.P. 66629, Nuevo León, Mexico; linda@cicese.mx; 3 Catedras CONACYT, Advanced Materials Research Center (CIMAV-Durango), CIMAV 110 Street, Ejido Arroyo Seco, Durango C.P. 34147, Durango, Mexico

**Keywords:** biocomposite materials, bioadsorbents, fluoride adsorption, drinking water, zirconium

## Abstract

The development of biocomposite materials used as adsorbents to remove ions in aqueous media has become an attractive option. The biomasses (base materials) are chemically treated and impregnated with metal cations, becoming competitive for fluoride-capture capacity. In this research, Valence orange (*Citrus sinensis*) and Red Delicious apple (*Malus Domestica*) peels were modified by alkaline treatment, carboxylation, and impregnation with zirconium (Zr). These materials were characterized morphologically and structurally to understand the modifications in the treated biomasses and the mechanism of fluoride adsorption. The results show changes in surface area and composition, most notably, an increment in roughness and Zr impregnation of the bioadsorbents. After batch experimentation, the maximum capacity of the materials was determined to be 4.854 and 5.627 mg/g for the orange and apple peel bioadsorbent, respectively, at pH 3.5. The experimental data fitted the Langmuir model, suggesting that chemisorption occurs in monolayers. Finally, the characterization of the bioadsorbents in contact with fluoride allowed the replacement of OH species by fluoride or the formation of hydrogen bonds between them as an adsorption mechanism. Therefore, these bioadsorbents are considered viable and can be studied in a continuous system.

## 1. Introduction

Globally, concentrations of fluoride ions (F^−^) in groundwater intended for human consumption exceed 1.5 mg/L [1,2], which is the maximum permissible limit (MPL) indicated by the World Health Organization (WHO). Millions of people ingest this kind of water, which increases the risk of dental and skeletal fluorosis reproductive and neurological problems, among others [1,2,3]. Usually, the separation of fluoride from water intended for human consumption can be carried out by precipitation/coagulation, ion exchange/adsorption, and membrane-based processes [4,5].

Although it is practical to remove high concentrations of fluoride, due to the energy demand, leaching of compounds in the treated water, and the generation of significant amounts of waste, coagulation precipitation methods are not currently a sustainable alternative for drinking water [4,6]. Membrane-based processes such as osmosis, ultrafiltration, and dialysis are well-known for being effective in a wide range of contaminant concentrations. However, the fouling and obstructions in the membranes and the operating costs are still a problem, and it is a challenge to apply them in a real matrix [4,5,7,8]. Finally, adsorption stands out as a method of separating contaminants in an aqueous medium, due to a wide range of adsorbent materials, as well as the requirement of lower investments and operating costs. In addition to the regeneration and reuse of the adsorbent, it enables the reduction of waste [4,6].

Among the fluoride-adsorbent materials, metal oxides and hydroxides (clays and zeolites), and bioadsorbents (BADs) (activated carbon, algae, chitosan, forestry, and agro-industrial residues) have been studied. Mainly, bioadsorption is of great interest to the scientific community due to the abundance of biomaterials and their low cost. Most of them are composed of biopolymers rich in functional groups, with high chemical stability, reactivity, and selectivity to the contaminant [4,9,10,11]. However, many BADs must be modified physicochemically to achieve high removal rates. Preparation methods frequently include biomass calcination, impregnation with metal oxides/hydroxides, and alkaline and acid treatments, allowing the purification and elimination of impurities in the base materials.

Biocomposite materials (BCMs) from modified waste biomasses used as BADs for fluoride removal have achieved efficiencies that compete with higher-cost materials and processes [4,11]. Among other promising candidates for BCMs as BADs, fruit peels stand out for their content of polymers rich in functional groups, such as polyphenols, hemicellulose, lignin, pectin, and cellulose [4,9]. In particular, Valencia oranges and Red Delicious apples are fruits consumed worldwide during all seasons, discarding tons of their peels [12,13,14], which are abundant in macromolecules [14,15,16]. Through alkaline treatment and carboxylation processes, the biomasses are induced to remove unnecessary compounds, transform cellulose, and increase the number of ligands, leading to an increment in the adsorption capacity of the pollutant of interest [17,18,19]. Besides, another current modification is based on supporting metal cations on biomasses [18,20,21,22,23,24]. Zirconium (Zr) has become a helpful material in environmental applications because it is inexpensive, non-toxic, chemically inert, and insoluble in water over a wide pH range [25,26,27]. Moreover, hydrated and polymerized Zr forms tetra and octonuclear ions, with electrical affinity and selectivity for fluoride, forming stable compounds [18,26,28].

In this research, orange peels (OP) and apple peels (AP) were modified by alkaline treatment, carboxylation, and Zr impregnation. The developed biocomposites were evaluated for fluoride removal and characterized to determine the changes undergone at each stage.

## 2. Materials and Methods

### 2.1. Materials and Reagents

In this work, the following reagents were used: calcium hydroxide (Ca(OH)_2_), sodium fluoride (NaF; JT Baker^®^, Radnor, PA, USA), zirconium oxychloride (Cl_2_OZr∙8H_2_O), and chloroacetic acid (ClCH_2_COOH; Sigma Aldrich^®^, St. Louis, MO, USA), sodium hydroxide (NaOH; Fermont^®^, Apodaca, Mexico), and hydrochloric acid (HCl; Fermont^®^ Monterrey, Mexico). The fresh fruit of Valencia oranges (*Citrus sinensis*) and Red Delicious apples (*Malus domestica*) were obtained from a local market in the city of Durango, Dgo. México. The fruit was washed with soap and tap water to remove agrochemical residues and dust. The peels of each fruit were removed with a knife.

The groundwater belongs to the aquifer Valle del Guadiana, located in Durango, Dgo, Mexico. The water sample was collected in high-density polypropylene containers, which were preserved at 4 °C until its characterization in physicochemical parameters (following the Standard Methods for Examination of Water and Wastewater APHA, AWWA, WEF).

Fluoride quantification was performed by the ion-selective method (ISE) in a Thermo Scientific Orion Versa Star^®^ (Apodaca, Mexico) with TISAB II ionic adjuster (Chelmsford, MA, USA) in a 1:1 ratio. Calibration was performed with 1.2 and 10 mg/L standards, and the slope was maintained at −54 to 60 Mv. By using inductively coupled plasma spectroscopy (ICP, iCAP 6500 Spectrometer Thermo Scientific^®^, Apodaca, Mexico) Zr was quantified in groundwater before and after treatment with the composite bioadsorbents.

### 2.2. Preparation of the Composite Bioadsorbents

The biomasses (OP and AP) were dried in a solar-thermal wind tunnel-type dryer for 5 h at ~40 °C. The dried peels were ground in an electric knife mill and sieved to a size between 250 and 500 µm. Powders of OP and AP were washed with deionized water to remove water-soluble compounds. Powders of OP and AP were alkaline-treated by adding deionized water and calcium hydroxide and the pH was adjusted to ~12. Subsequently, carboxylation was performed by immersing the alkalinized powders of OP and AP with chloroacetic acid, adjusting the pH to between 8 and 10. Finally, the biomasses were loaded with Zr by adding Zr oxychloride, adjusting the pH to ~2. In each treatment, constant agitation was maintained for 24 h, and pH adjustments were made with sodium hydroxide lentils. At the end of each step, the biomasses were washed with plenty of deionized water and dried in the sun and at room temperature (~30 °C) for 24–48 h. The final products are named BOP-Zr and BAP-Zr, being B for bioadsorbent. Figure S1 summarizes graphically the preparation of the bioadsorbent materials.

### 2.3. Characterization of Composite Bioadsorbents

To relate the physicochemical properties and transformations undergone by the bioadsorbents, samples of the biomasses (OP and AP) and the bioadsorbents (BOP-Zr and BAP-Zr) were subjected to different characterization techniques, before and after contact with the water to be treated. 

The bioadsorbents were analyzed for N_2_ physisorption (77.35 K), specific area by the Brunauer, Emmett, and Teller (BET) method and pore size distribution (Quantachrome Autosorb 1-C apparatus^®^, Boynton Beach, FL, USA). Material size, composition and morphology were obtained by scanning electron microscopy with energy dispersive X-ray spectroscopy (SEM-EDX) in a Nova NanoSEM 200 FEI^®^ (Thermo Fisher Scientific^®^, Hillsboro, OR, USA) with a current 0.31–0.63 nA and a voltage of 15.0 kV. The presence of functional groups was verified by Fourier transform infrared transmission spectroscopy (FTIR) in a Nicolet iS50 Thermo Scientific^®^ spectrometer (West Palm Beach, FL, USA), in a measurement range of 4000 to 400 cm^−1^. The thermal stability of the samples was studied by means of a thermogravimetric analysis-differential scanning calorimetry (TGA/DSC, TA Instrument^®^, New Castle, DE, USA) in N_2_ atmosphere under a flow rate of 30 mL/min and a heating rate of 10 °C/min, varying the temperature from room temperature to 1000 °C in an Al_2_O_3_ crucible. To determine the crystalline nature and the composition, X-ray diffraction (XRD) analysis was carried out (Panalytical Empyream diffractometer^®^, Malvern, Worcestershire, UK), with Cu Kα radiation, data were collected in the 2θ range of 5–70°. The chemical state of the elements present was obtained by X-ray photoelectron spectroscopy (XPS) in an Escalab 250Xi XPS Spectrometer by Thermofisher^®^ (East Grinstead, West Sussex, UK) with Al Kα source. The spectra were corrected to the carbon C1s signal at 284.8 eV, the analysis radius is 650 μm and the charge compensation gun was used to avoid signal drift.

### 2.4. Batch Adsorption Experiments

The adsorption of fluoride was studied at a concentration of 4 mg/L and at pH 3, 4, 5, and 6 (adjusted with sodium hydroxide and hydrochloric acid 0.1 M), for 24 h of contact time. To describe the adsorption capacity and type of adsorption of BOP-Zr, and BAP-Zr, NaF solutions were prepared, in the range of 2–10 mg/L. The pH was not adjusted, because the bioadsorbents modify the pH of the working solution, which was kept at ~3.5 ± 0.2. The contact times were 1, 3, 5, 8, 24, and 48 h. The percentage and amount of fluoride removed (mg/g) were calculated by Equations (1) and (2) respectively,

(1) $$\%\;\mathrm{Removal}=\frac{C_{i}-C_{f}}{C_{i}}\times 100$$

(2) $$Q=\frac{C_{i}-C_{f}}{m}\times V$$

where C_i_ and C_f_ refer to the initial and final fluoride concentrations (mg/L), m is the mass of bioadsorbent used (g), and V is the volume of fluoridated water used (mL). The effect of co-existing ions, such as arsenate (As^5+^), sulfate (SO_4_^2−^), carbonate (HCO^3−^), phosphate (PO_4_^3−^), nitrate (NO_3_^−^), and chloride (Cl^−^) was determined by adding different amounts of Na_2_HAsO_4_, Na_2_SO_4_, NaHCO_3_, NaH_2_PO_4_, NaNO_3_, and NaCl salts to a 4 mg fluoride/L solution, accordingly, for a contact time of 24 h and letting the pH of the reaction medium remain as is. The fluoride removal capacity of the bioadsorbents was tested in a natural matrix (well water), and the pH adjustment was performed with hydrochloric acid up to ~3.5. The contact times were set at 1 and 24 h. Throughout the described experimentation, the dosage of biocomposite was 1 g/L. To reach equilibrium, constant stirring at 320 RPM and room temperature ~30 °C was carried out in an IKA KS 130 basic orbital shaker^®^ (Wilmington, NC, USA).

## 3. Results

### 3.1. Surface, Morphological, and Structural Characterization of the Adsorbent Materials

#### 3.1.1. BET

The surface area (m^2^/g) was determined to be 0.751 for BOP-Zr, and 0.521 for BAP-Zr. So, the volume (cm^3^/g) and pore size (nm) for BOP-Zr was estimated at 0.00141 and 6.07, respectively, whereas BAP-Zr had values of 0.00066 cm^3^/g and 5.06 nm Table S1 includes a summary of the results obtained in the BET analysis. By pore size, according to IUPAC (International Union of Pure and Applied Chemistry), both bioadsorbents are classified as mesoporous [29], a frequent feature in fluoride adsorbent materials [30,31,32]. Because the radius of the fluoride anion is 0.133 nm [33,34,35] and that the pore size of both bioadsorbents is significantly more prominent, it is possible that fluoride adsorption [34,35] takes place in three well-defined stages. First, fluoride anions’ mass transfer to the bioadsorbent surface (molecular diffusion). Then, adsorption happens on the surface of the bioadsorbent particles. Finally, we have an intraparticle distribution, where fluoride is adsorbed on the inner surface of the bioadsorbent particles [36,37].

**Figure 1:**
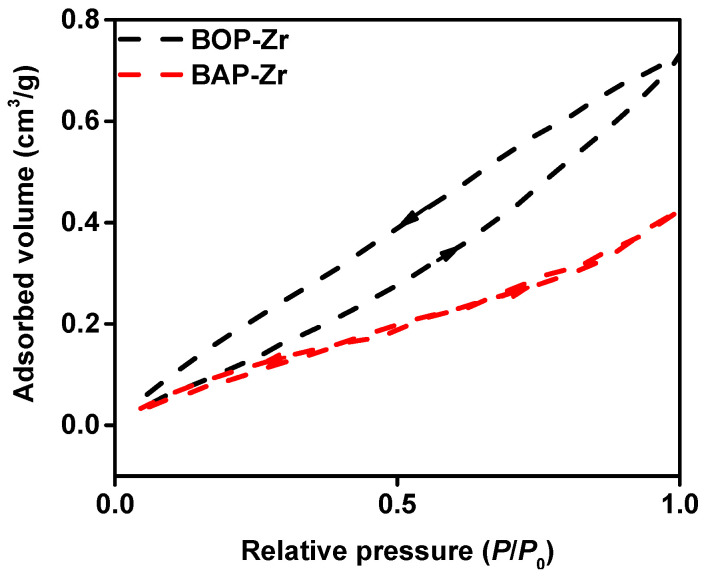
N_2_ adsorption-desorption isotherms of the bioadsorbents BOP-Zr (black-dashed line) and BAP-Zr (red-dashed line).

Figure 1 shows the N_2_ adsorption–desorption isotherms of the bioadsorbents, in both cases, corresponding to type IV, confirming mesoporosity. Both samples show hysteresis loops [29,32] associated with pore condensation and the cavities’ size, distribution, and geometry [29]. On the one hand, BOP-Zr presents an H2 hysteresis. This kind of material does not have a specific shape, and the pore distribution is not defined [29,32]. On the other hand, BAP-Zr can be seen as an H4-shaped hysteresis, indicating pores with a narrow-slit form and containing micropores inside. In both kinds of hysteresis loops, low-pressure conditions are observed, associated with non-rigid materials and pore swelling. Both open loops are related to chemical adsorption [29].

#### 3.1.2. SEM-EDX

**Figure 2:**
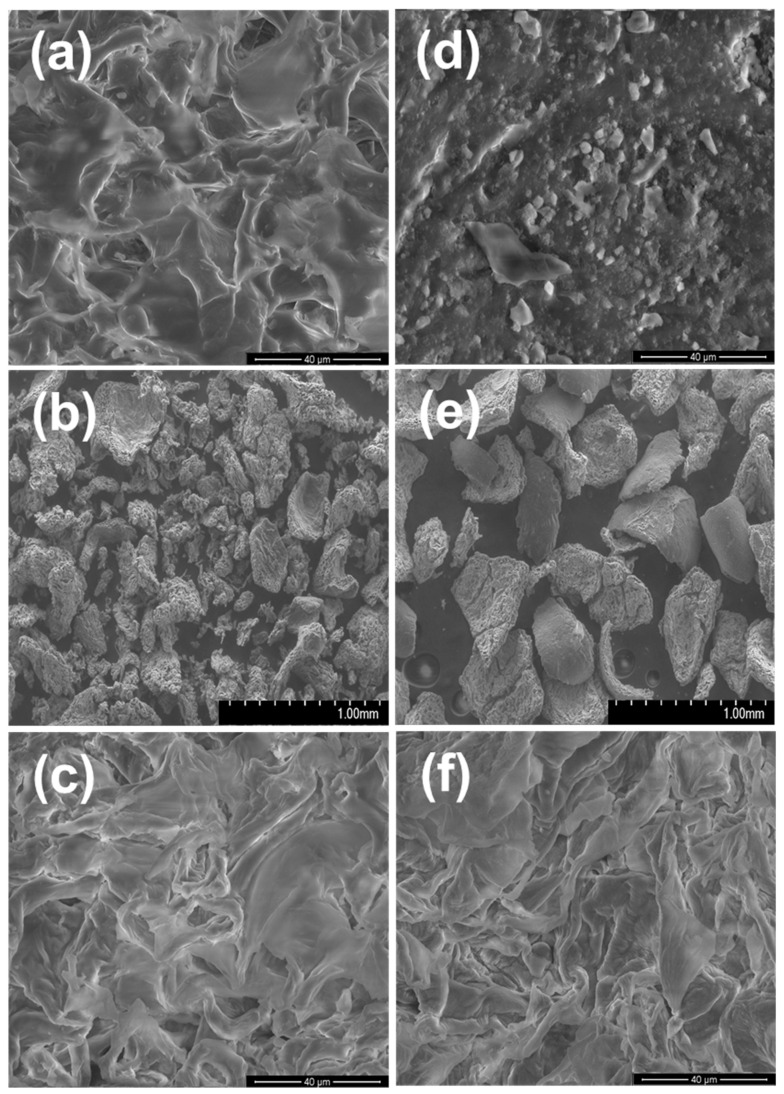
SEM micrographs of (**a**) OP 40 μm, (**b**) BOP-Zr 1000 μm, (**c**) BOP-Zr 40 μm, (**d**) AP 40 μm, (**e**) BAP-Zr 1000 μm, and (**f**) BAP-Zr 40 μm.

The texture and morphology of the peels and the bioadsorbents are observed by SEM characterization (Figure 2). Figure 2a,d show the images corresponding to OP and AP, respectively. A rough surface is distinguished for OP, whereas the superficial aspect of AP is smooth, with some flakes. A panoramic sight (1000 μm) of the bioadsorbents, represented in Figure 2b,e, exhibits that these materials consist of amorphous particles, attributed to the type of material and the method used for its size reduction. We compared the neat material with the bioadsorbents by doing a zoom (Figure 2c,f), showing rough surfaces in both cases.

It is essential to highlight the change in texture, which the materials undergo due to the chemical treatment. In OP, the folds increased in number and became finer, attributed to the decomposition and breaking of bonds in lignin and hemicellulose during the alkaline treatment; furthermore, this increases the roughness of the cellulose [38]. Regarding AP, it changed from a smooth surface to a rough surface, related to the alkaline treatment, which increases the roughness in the material [17]. The increase in roughness on the surface of both bioadsorbents favors the contact between the fluoride and the active sites [39,40]. The average size of BOP-Zr is 80 µm, and for BAP-Zr, 388 µm.

Also, we performed an analysis for the EDX composition on the samples, belonging to orange and apple peels, without any treatment shown. Both are mostly (wt.%) composed of C and O, and Zr presence is not detected. Table 1 shows the average composition in % weight of each of the materials studied. As expected, the highest percentages of both materials correspond to C and O, which is the organic nature of the materials. In addition, Zr is present at 30% for BOP-Zr and 22% for BAP-Zr, indicating that the organic matrix permeates or supports Zr.

### 3.2. Composition Characterization of the Absorbents

#### 3.2.1. FTIR

**Figure 3:**
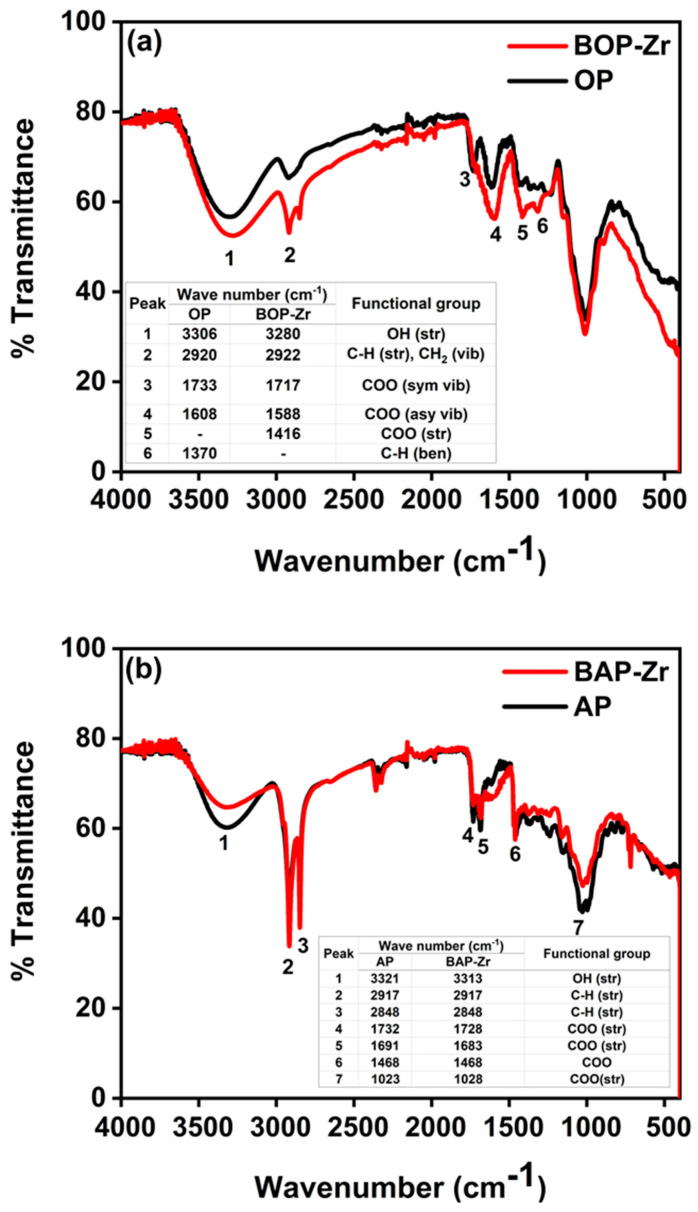
FTIR-ATR spectra of (**a**) OP and BOP-Zr, (**b**) AP and BAP-Zr.

The FTIR-ATR spectra for OP and AP samples, as well as in their respective bioadsorbents, are shown in Figure 3. Figure 3a presents OP and BOP-Zr spectra, and the results for AP and BAP-Zr samples are displayed in Figure 3b. The functional groups identified in the spectra are summarized in the tables attached to its corresponding spectrum.

On the one hand, the FTIR in Figure 3a confirms the -OH groups of polymeric compounds such as cellulose, pectin, and carboxylic acid [41]. Especially in BOP-Zr, the peak assigned to carboxylic acid was slightly shifted concerning the OP peak (3280–3306 cm^−1^). The BOP-Zr peaks at 2922 and 1717 cm^−1^ and OP peaks at 2920 and 1733 cm^−1^ correspond to the stretching of the C-H and COO groups, respectively [42,43]. Also, the OP peak at 1733 cm^−1^ presents a shift to a lower frequency (1717 cm^−1^) in BOP-Zr, due to the increased molecular weight of the charged metal ion in the biomass [41].

On the other hand, the FTIR spectra for AP and BAP-Zr (Figure 3b) contain a broadband at 3321 and 3313 cm^−1^, corresponding to the OH bond of the polyphenols. The sharp peaks in BAP-Zr at 2917 cm^−1^ and 2848 cm^−1^ correspond to the C-H stretching of aliphatic compounds [21]. The peak at 1732 cm^−1^ for the AP sample shifts to 1728 cm^−1^ in the BAP-Zr spectrum, indicating a product of Zr loading on the bioadsorbent [41] and the presence of COO of the ester group [26,44]. The change on the peaks, from the biomass spectra to these of the bioadsorbents, is evidence of the chemical modification that the peels underwent in the BOP-Zr and BAP-Zr preparation process [45].

#### 3.2.2. TGA

**Figure 4:**
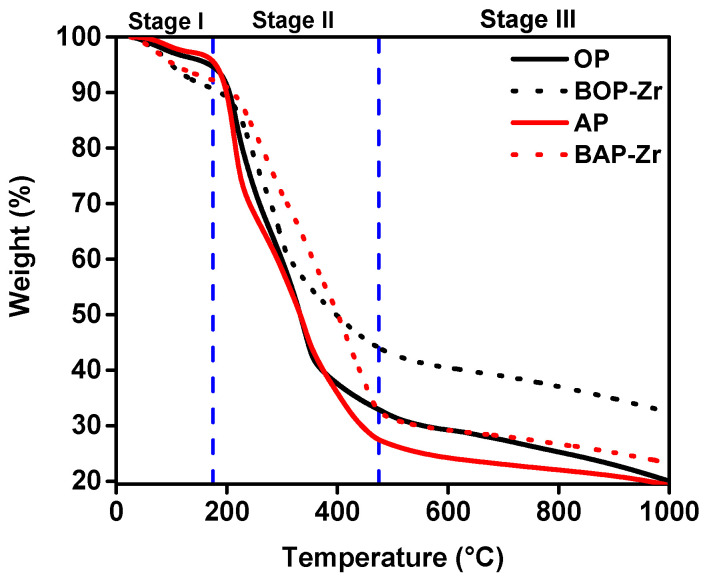
TGA curves of weight loss rate for OP (black line), BOP-Zr (black dashed-line), AP (red line), and BAP-Zr (red dashed-line) samples.

The thermogravimetric curves for the peels and their respective bioadsorbents are presented in Figure 4. In both cases, the biomasses (solid lines) and their bioadsorbents (dashed lines) show the weight-losing steps characteristic of lignocellulosic compounds [46], represented by three stages. The first one (25–175 °C) is attributed to the loss of water (moisture) and other volatile substances [18]. Stage II takes place between 175 and 475 °C, which is related to the simultaneous loss of cellulose (250–420 °C) [46], pectin (<400 °C) [47], lignin 300–550 °C [18], and hemicellulose (220–315 °C) [48], essential constituents of OP and AP [14,16,44,49]. The chemical decomposition of hemicellulose and lignin during alkaline treatment provides more excellent thermal stability to BOP-Zr and BAP-Zr, as observed in the increment of degradation temperatures in stage II [50]. Finally, the decomposition of carbonaceous residues comes about in the last stage above 475 °C [18].

The loss of water molecules in stage I was higher in BOP-Zr (9%) and BAP-Zr (8%) concerning their respective biomasses (5% for both OP and AP samples). Consequently, the water loss at a lower temperature can also be related to the surface modifications of the materials during alkaline treatment [50]. Also, the reduction in BOP-Zr (56%) and BAP-Zr (68%) weight loss, compared to OP (67%) and AP (73%) in stage II, is the result of the remotion of hemicellulose and lignin through the alkaline treatment [45,51]. Finally, in stage III, the weight gained in both inorganic wastes is the consequence of Zr impregnation [18].

#### 3.2.3. XRD

**Figure 5:**
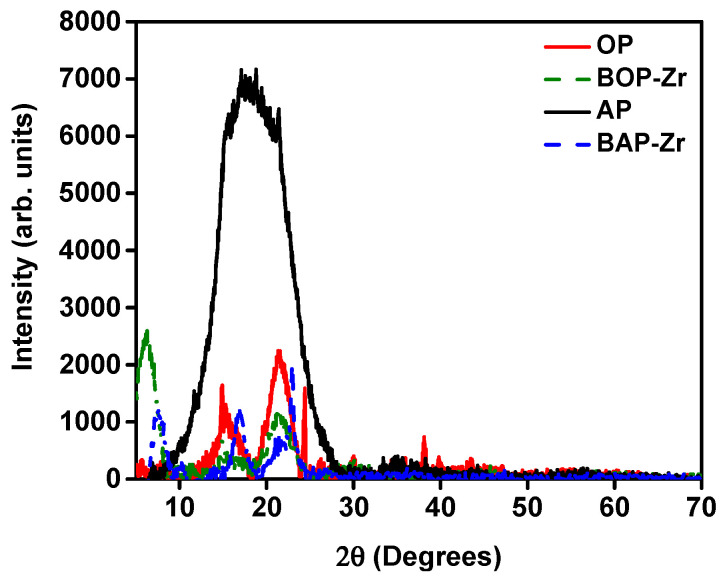
Diffraction pattern, OP (red line) and BOPZr (green-dashed line), AP (black line) and BAPZr (blue-dashed line).

The diffraction analysis for all kinds of samples is shown in Figure 5. Except for the AP sample, XRD results are associated with amorphous or semi-amorphous polymeric materials [52]. On the one hand, the OP pattern presents a large amorphous zone, with a peak within the range of 20–25°, resulting from amorphous materials such as hemicellulose and lignin. The peaks found at ~15° and 22° are associated to amorphous cellulosic material [43,53,54]. On the other hand, the XRD study for AP (Figure 5) presents a broad peak at 18°, which is related to the fibrous and non-crystalline constitution of the sample [13].

For the bioadsorbent samples, diffraction patterns are presented. The BOPZr and BAP-Zr patterns, with baseline subtraction, show amorphous materials, with peaks at 2θ = 15°, 22°, and 35° for BOP-Zr and 2θ = 16.92°, 23°, and 35° for BAP-Zr. Thus, we identified the presence of type I crystalline cellulose, which is prevalent in plant tissues [19,53,54]. The changes between patterns OP/BOP-Zr and AP/BAP-Zr patterns confirm, as identified by SEM analysis that the alkaline treatment removed compounds such as hemicellulose and lignin with cellulose persisting in a higher ratio [38].

Additionally, for BOP-Zr and BAP-Zr, the presence of Zr(OH)_4_ and polymerized species, meaning Zr^4+^, is associated [18,55]. In 2010, Mulinari reported that, under acidic conditions, a donor–acceptor bond (Lewis acid) is formed between Cl_2_OZr∙8H_2_O and the O of the C bonds present in cellulose. When Zr is associated with the biopolymer, a reduction in peak size is observed between ~15° and 22° (2θ), attributed to cellulose, representing the amorphous character of Zr-OH [53]. OP and BOP-Zr diffractograms show peak reduction, confirming the successful impregnation of Zr^4+^ into OP. The Zr^4+^ loaded in the biomass is transformed into an amorphous phase, composed of polymerized and hydrolyzed species. Through the incorporation of water molecules and OH^−^ species, they are forming tetranuclear [Zr_4_(OH)_8_(H_2_O)16]^8+^ and octonuclear [Zr_8_(OH)_20_(H_2_O)_24_]^12+^ ions. The abundance of OH^−^ ions promotes ligand exchange with fluoride; thus, its removal from the aqueous medium [28,55]. Due to its electrical affinity, Zr^4+^ has a high selectivity for fluoride ions, forming stable compounds [16]. Therefore, the presence of Zr^4+^ in bioadsorbents is a positive and helpful result to explain the mechanism by which fluoride removal from treated water occurs.

### 3.3. Fluoride Removal by Bioadsorbents

#### 3.3.1. Influence of pH

**Figure 6:**
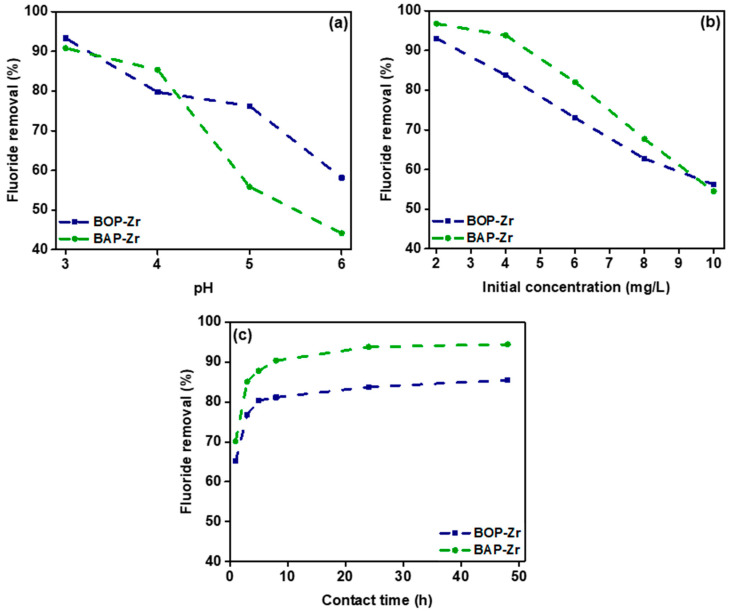
Efficiency of the fluoride removal by the bioadsorbents in function of: (**a**) pH; (**b**) the initial concentration, mg/L; and (**c**) contact time, h.

The influence of pH on fluoride removal by the BOP-Zr and BAP-Zr samples at different concentrations is observed in Figure 6a. The two bioadsorbents showed a special removal rate when the pH was 3 and 4. The maximum removal rates reached ~90% at a pH value of 3. Therefore, we established the optimum working pH for both samples at this value, similar to those used in the literature for biocomposite materials [22,42,49,56]. The polymers, of which modified biomasses are made up, have large functional groups with a pH that may influence the electrical charge of the bioadsorbent surface and the ionization of the adsorbing species, leading to this removal rate condition [23]. Nevertheless, the surface of the bioadsorbent is positive when the pH is low, making fluoride adsorption easier [56]. Moreover, it is known that fluoride adsorption at a pH lower than 3 is not viable due to the formation of hydrofluoric acid (HF) [18,23,28,41,49].

It is important to note that the removal capacity decreases as pH increases. For instance, at pH 6, F removal is less than 60%. The decrement in removal rates is attributed to the competition of hydroxyl species with the F ions for the coordination sites of the Zr loaded onto the biomasses [28,49]. Then, an increment in pH leads to a negative charge on the surface of the bioadsorbents, repelling the fluoride ions [11]. Finally, it is observed that the pH at the end of treatment with the two bioadsorbents increases slightly due to the replacement of OH species instead of F ions [16].

#### 3.3.2. Influence of the Initial Fluoride Concentration

The ratio of fluoride removal to that of the initial fluoride concentration was determined by using different concentrations at 2, 4, 6, 8, and 10 mg/L, at a contact time of 24 h. Figure 6b shows a decrement of the fluoride removal as the concentration of initial fluoride increases, which is the consequence of the saturation of the coordination sites of Zr [18,56]. The maximum capacity of the bioadsorbents at 10 mg/L concentration was close to 55%. For the lowest concentrations tested at 2 and 4 mg/L, 84–97% removals were achieved. The removal rates of BAP-Zr are slightly higher than those of BOP-Zr.

#### 3.3.3. Influence of Contact Time

The removal of fluoride (4 mg/L) by BOP-Zr and BAP-Zr was studied at contact times ranging from 1–48 h. Figure 6c presents the dynamics of the removal phenomenon. It displays that the process rates increase rapidly in the first 8 h of contact time. However, the highest removal rate occurs during the first hour, where 65 and 70% fluoride removal is achieved for BOP-Zr and BAP-Zr, respectively. The affinity of BOP-Zr and BAP-Zr is evident due to the high removal rates during the first 5 h of contact time [24]. After 12 h of contact, the removing process is no longer significant, related to the initial fluoride concentration gradient and the number of active sites at the beginning of the process [18]. Therefore, the optimal contact time was determined to be 5 h for both bioadsorbents.

#### 3.3.4. Adsorption Type and Capacity

**Figure 7:**
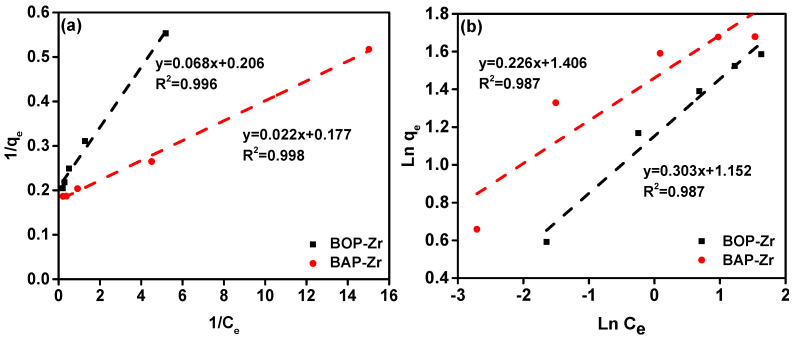
Adsorption isotherms for the bioadsorbents by using the models of (**a**) Langmuir, and (**b**) Freundlich. The black and red dashed-lines represent their respective linear fit.

Experiments were carried out to determine the amount of fluoride absorbed by the bioadsorbent. According to the Langmuir and Freundlich isotherm models, the data obtained were fitted. These models allow us to determine the character of the adsorption (physical or chemical) and the formation of mono- or multilayers [18]. Figure 7 illustrates the Langmuir (a) and Freundlich (b) adsorption isotherms obtained for BOP-Zr and BAP-Zr. The parameters calculated by using each model are shown in Table 2. According to the literature, the q_max_ of BOP-Zr (4.854 mg/g) and BAP-Zr (5.627 mg/g) for biomasses reported for fluoride removal is higher than other kinds studied. Therefore, our materials can be considered viable and promising for removing fluoride ions.

After calculating the coefficients of determination (R^2^) for both models, we found that the Langmuir model is higher than that of the Freundlich isotherm. We implied that the experimental data describe with greater certainty the chemical type of adsorption, with the formation of adsorbate monolayers on the surface of the bioadsorbent. To determine the feasibility of the model, the Langmuir separation factor (RL) was determined by using the following equation:

(3) $$\mathrm{RL}=\frac{1}{1+K_{L}C_{i}}$$

where C_i_ is the initial concentration of adsorbate in solution (fluoride). The RL value determines whether the adsorption is linear (RL = 1), favorable (0 < RL < 1), unfavorable (RL > 1), or irreversible (RL = 0) [12,26]. The RL values of a C_i_ = 4 mg/L indicate favorable adsorption for both BOP-Zr and BAP-Zr.

#### 3.3.5. Co-Existing Ions

**Figure 8:**
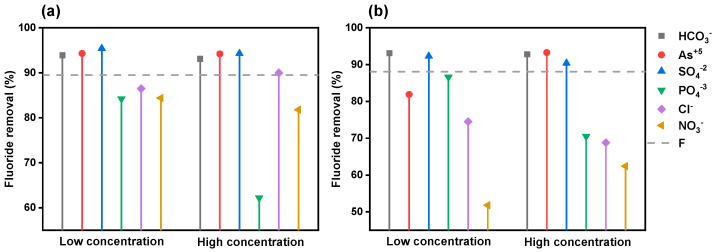
Fluoride removal in the presence of other ions (**a**), BOP-Zr (**b**) BAP-Zr, with F_initial_ = 4 mg/L, Contact time 24 h and pH 3.5 ± 0.2.

To determine the selectivity of the bioadsorbents, fluoride adsorption in the presence of other ionic species present in groundwater was tested. Table S3 shows the physicochemical characterization of the groundwater used in the experiment to determine the adsorption of fluoride ions in a real matrix. The collected water belongs to the well “San Luis” (24.05587778, −104.59307222), located in Durango City, Dgo. Mexico. Figure 8 represents the results of this study at low and high concentrations.

It is observed that both BOP-Zr and BAP-Zr have a decrement in the fluoride-removing process in the presence of phosphate, chloride, and nitrate ions. This decreasing event is more pronounced for the BAP-Zr samples. The phenomenon is exacerbated at high ion concentrations. Whereas phosphate ions negatively affect the adsorption of fluoride by BOP-Zr in this scenario, nitrate ions do not constitute significant interferents. Typically, phosphates and nitrates are not present in high concentrations in water intended for human consumption, but an increase in their concentration refers to the contamination of the supply source [57,58]. Moreover, as they are not part of most of the ions in natural waters, their effect does not interfere with the potential of BOP-Zr and BAP-Zr. In a natural matrix (well water), the removal percentages were higher than those of solutions prepared in the laboratory, because of the deionized water-leaching elements from the adsorbent material.

In contrast, in the case of well water, ionic species such as Ca^2+^ may contribute to fluoride removal. Therefore, the selectivity of BOP-Zr is phosphate > nitrate > chloride > chloride > sulphate > arsenate > bicarbonate, whereas the same process for BAP-Zr is nitrate > chloride > phosphate > sulphate > arsenate > bicarbonate. Nevertheless, BOP-Zr and BAP-Zr are not affected in the presence of bicarbonate species, as described in other similar materials [18,22,42]. These results confirm the affinity of both bioadsorbents for fluoride.

#### 3.3.6. Fluoride Removal in Well Water

Additionally, fluoride removal in laboratory solutions and well water samples was studied under the same conditions. Table 3 contains the removal percentages achieved in well water and laboratory solutions by the bioadsorbents under study. The experimentation showed that BOP-Zr and BAP-Zr remove fluoride at a contact time of 1 h, below the MPLs established by the WHO. This was achieved by lowering the fluoride concentration from 3.89 mg/L to 0.58 for BOP-Zr, and 1.20 for BAP-Zr. The bioadsorbents earned 85 and 69% removal at 1 h and reached the maximum reductions at 24 h of 95 and 93% for BOP-Zr and BAP-Zr, respectively. The rapid adsorption at the beginning of the contact is due to the availability of the active sites. After 24 h, the removal rates represent the equilibrium reached saturation of such active sites. The difference in the removal rates between the bioadsorbents is related to the number of coordination sites available in BOP-Zr.

It is important to note that the two bioadsorbents remove fluoride and arsenic (As) simultaneously, but the latter’s removal reached 80%through 24 h. Finally, the initial concentration of As in the treated water is safe according to the WHO MPL, set at 0.010 mg/L. The adsorption of fluoride was found to be similar between the treated water types, which means that the presence of anionic species typical of groundwater does not decrease the adsorption capacity of fluoride ions and As of both materials.

Finally, Zr was quantified by ICP in the treated well water, taking the same untreated well water as a reference. We demonstrate the stability of both bioadsorbents because Zr was not detected in the order of mg/L in any sample. In other words, there is no leaching of Zr in the treated water [16].

#### 3.3.7. Adsorption Mechanism

The interactions between adsorbent and adsorbate can be elucidated by checking the changes in the XPS spectra of the bioadsorbents, before and after fluoride capture.

All the results for the three deconvolution processes (C1s, O1s and Zr3d) are summarized in Table S3.

**Figure 9:**
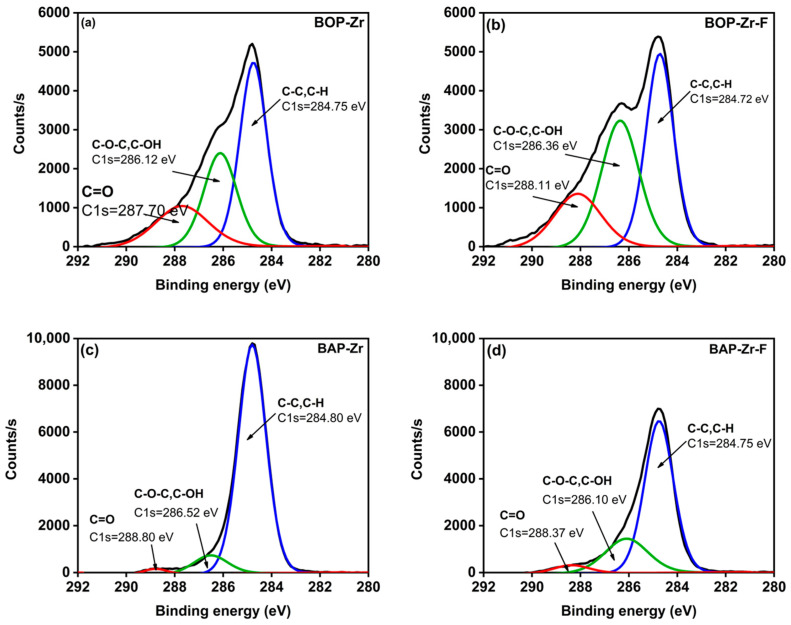
Deconvoluted XPS spectra for C1s: (**a**) BOP-Zr, (**b**) BOP-Zr-F, (**c**) BAP-Zr, and (**d**) BAP-Zr-F.

The results of the deconvolution of the XPS spectra for the C1s are presented in Figure 9. Before fluoride capture, in both BOP-Zr (Figure 9a) and BAP-Zr (Figure 9c), the contribution of C-C/C-H, C-O-C/C-OH, and C=O is observed. After fluoride adsorption, there are leftward shifts in the C-O-C/C/C-OH and C=O peaks of BOP-Zr-F. This shift toward higher binding energy is related to hydrogen bonding with the fluoride and OH groups. These groups are replaced with fluoride ions, forming C-F bonds. Also, the shift is associated with the electrostatic interaction between fluoride and protonated carbonyl groups [59]. In BAP-Zr-F, a significant decrease in the area of the C-C/C/C-H peak is observed (from 14,292 to 9897 CPS.eV), indicating an ion exchange between the fluoride and the functional group [60]. Moreover, the C-O-C-C/C-OH and C=O peaks become broader, from 1430 and 309 to 3315 and 675 CPS.eV, respectively. Moreover, there is a shift in the binding energy to the right due to the interaction of fluoride with these groups [60].

**Figure 10:**
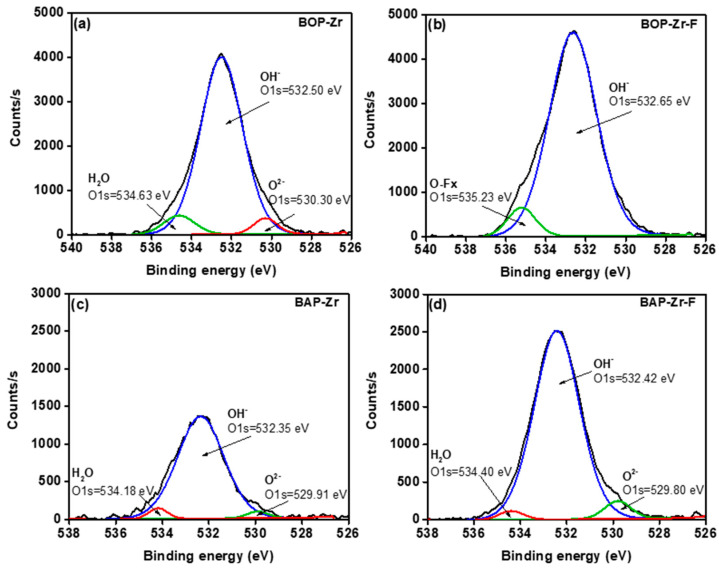
Deconvoluted XPS spectra for O1s, (**a**) BOP-Zr, (**b**) BOP-Zr-F, (**c**) BAP-Zr, and (**d**) BAP-Zr-F.

The respective deconvolution for O1s, in BOP-Zr and BAP-Zr spectra, are shown in Figure 10. The XPS curve present three representative peaks. The first one, located in the binding energies (eV) 530.30 and 529.91 for BOP-Zr and BAP-Zr, respectively, is associated with the O^2−^ of Zr of the metal oxide (M-O). The second peak is located at 532.50 for BOP-Zr and 532.35 for BAP-Zr, assigned to the hydroxyl group (OH^−^) of Zr. Finally, the binding energies (eV) 534.63 (BOP-Zr) and 534.18 (BAP-Zr) are attributed to the adsorbed water (H_2_O) [61,62,63]. The bioadsorbents that had contact with fluoride showed important changes in the peaks that constitute the O1s signal. Regarding BAP-Zr, the areas of the M-O and M-OH peaks decreased significantly, which suggests the role of O^2−^ and OH^−^ in the fluoride adsorption [62]. For the BOP-Zr bioadsorbent, only the M-OH peak had that significant increase, which involves the participation of this species in the fluoride capture. However, the O^2^^−^ and H_2_O peaks disappear, and the peak associated with O-Fx become visible.

**Figure 11:**
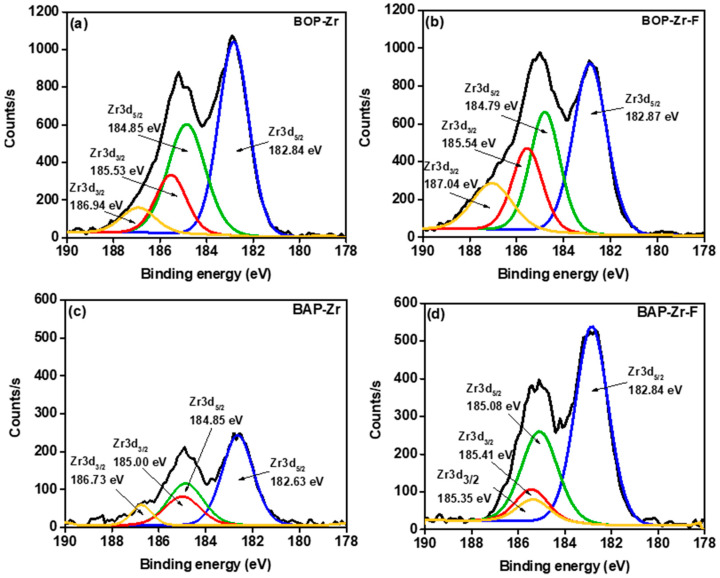
Deconvoluted XPS spectra for Zr 3d, (**a**) BOP-Zr, (**b**) BOP-Zr-F, (**c**) BAP-Zr, and (**d**) BAP-Zr-F.

The deconvoluted XPS spectra for Zr3d in BOP-Zr and BAP-Zr, before and after fluoride contact, are shown in Figure 11. The spectra of BOP-Zr and BAP-Zr present Zr3d_5/2_ and Zr3d_3/2_ doublets, indicating the presence of Zr^4+^ [64,65]. The Zr3d_5/2_ peaks with binding energies 182.84 and 182.63 for BOP-Zr and BAP-Zr, respectively, confirm the presence of ZrO_2_ [66].The binding energies 184.79 and 187.04 of the Zr3d_5/2_ and Zr3d_3/2_ peaks in BOP-Zr-F have been associated with ZrF_4_ [66,67,68,69]. In BAP-Zr-F, the Zr3d_5/2_ doublet shows a left shift (increase) in binding energy and a broadening of the peaks relative to BAP-Zr. Dou et al., in 2012, attributed these changes to the formation of new zirconium species, including ZrF_4_ and Zr-oxyfluorides. When fluorine bonds to Zr, it subtracts electrons in Zr, increasing the binding energies in the Zr3d_5/2_ and Zr3d_3/2_ peaks. The more fluorine atoms that bind to Zr, the higher the Zr3d peak would shift to higher binding energy [66].

Pantano et al., 1988, associated the binding energies (E_b_) of Zr3d vs. the Pauling charge, q(P), which is calculated by summations of the Zr ligands and the electronegativities of fluorine and oxygen. Also, it was assumed that the E_b_ and q(P) relationship by using the ZrO_2_, ZrO_0.6_F_2.8_, and ZrF_4_ standards. Therefore, the formation of F-ZrO_2_ species is associated. We plotted the binding energy of Zr3d vs. Pauling charge in zirconium species (Figure S2). We found that the binding energy of Zr3d_5/2_ in BAP-Zr-F (182.84 eV) is related to the ZrO_3_F_4_ species.

**Figure 12:**
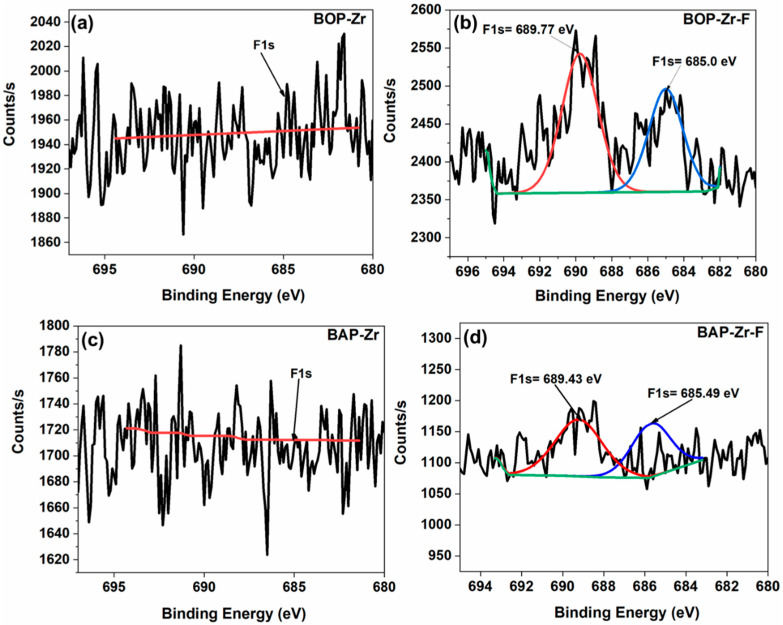
Deconvoluted XPS spectra for F1s: (**a**) BOP-Zr, (**b**) BOP-Zr-F, (**c**) BAP-Zr, and (**d**) BAP-Zr-F.

Finally, Figure 12 shows the deconvoluted XPS spectra for F1s: (a) BOP-Zr, (b) BOP-Zr-F, (c) BAP-Zr, and (d) BAP-Zr-F. In BOP-Zr and BAP-Zr, the presence of fluoride was not detected, whereas in the materials in contact with fluorinated water, the F1s peak appears. The deconvolution of the spectrum allows us to identify the presence of both ZrF_4_ and ZrO_3_F_4_, at binding energies of 685.0 eV and 689.77 eV for BOP-Zr-F, and 685.49 eV for ZrF_4_ and 689.43 eV for BAP-Sr-F, respectively [66,68,70,71,72]. These data are consistent with the Zr3d peak found through deconvolution, as mentioned before.

**Figure 13:**
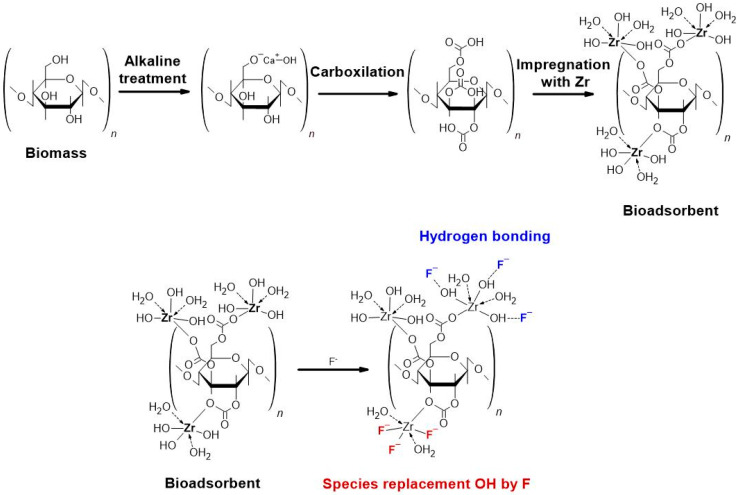
Synthetic route of zirconium loaded bioadsorbent and mechanism of fluoride adsorption.

The mechanism of fluoride adsorption for both bioadsorbents is proposed, shown in Figure 13. In this scheme, the modification of the biomasses to biocomposites is represented, and the adsorption of fluoride by the studied bioadsorbents is outlined. It is important to note that the different mechanisms occur simultaneously and not exclusively for each bioadsorbent. Figure 13 shows the exchange from OH to F in red color. The mechanism is similar to the publications of different authors [4,16,18,53]

## 4. Conclusions

After the physicochemical treatment of the base materials, two mesoporous bioadsorbent materials were successfully developed from Valence orange (*Citrus sinensis*) and Red Delicious apple (*Malus domestica*) peels.

The SEM and TGA analyses allow relating the changes in the surface of the materials, this due to the alkaline treatment; where the bioadsorbents suffered an increase in roughness, contact with the adsorbate was favored.

XRD and TGA confirmed the elimination of the lower molecular weight compounds, with cellulose prevailing to a greater extent, which is an excellent support matrix for the metal cation. The evidence confirms the incorporation of the polymerized Zr hydroxide into the base polymer. This Zr^4+^ is very fluoride affine and able to form stable compounds with fluoride.

XPS analysis showed that both BOP-Zr and BAP-Zr adsorb fluoride by forming bonds between the adsorbate and the adsorbent. The polymerized Zr is involved in the formation of these bonds, either by replacement of OH species by F or by hydrogen bonding.

The high selectivity of the materials was demonstrated, but no significant interferences with the rest of the ionic species (HCO_3_^−^, As^5+^, SO_4_^2−^, PO_4_^3−^, Cl^-^ y NO_3_^−^) studied were found. In addition, both materials are stable at the pH of study (3–6), because Zr was not detected in the treated water.

In conclusion, the morphological and surface characterization shows that both mesoporous biocomposites contain abundant cellulose supporting Zr^4+^, in the form of oxides or hydroxides. The BOP-Zr bioadsorbent reached a maximum capacity of 4.854 mg/g, whereas the BAP-Zr was 4.413 mg/g, being in both cases competitive with materials previously reported in the literature and promising for further study. Furthermore, these bioadsorbents are able to simultaneously remove fluoride and arsenic from groundwater sources, with minimum contact times of 1 h and pH = 3.

## Figures and Tables

**Table 1:** Average composition (% weight) of the materials under study.

Sample	Element (% Weight)				
	C	O	Ca	Cl	Zr
OP	55.72 ± 2.49	42.40 ± 2.44	0.41 ± 0.09		
BOP-Zr	34.68 ± 1.12	30.85 ± 2.55		1.36 ± 0.18	33.08 ± 2.95
AP	85.50 ± 0.47	14.51 ± 0.46			
BAP-Zr	43.30 ± 1.29	32.57 ± 1.11		2.01 ± 0.09	22.01 ± 0.62

**Table 2:** Summary of parameters for the Langmuir and Freundlich models obtained for the bioadsorbents.

Bioadsorbent	Isotherm Model	q_max_ (mg/g)	Constant	R^2^
BOP-Zr	Langmuir	4.854	K_L_ 3.043 (L/mg) RL 0.08	0.996
	Freundlich	3.297	K_F_ 3.165 (mg/g)	0.987
BAP-Zr	Langmuir	5.627	K_L_ 7.969 (L/mg) RL 0.03	0.998
	Freundlich	4.413	K_F_ 4.308 (mg/g)	0.987

**Table 3:** Removal percentages of fluoride (after 1 and 24 h) and arsenic (after 24 h) were achieved in well water and laboratory solution by BOP-Zr (C_f_ = 0.58 mg/L) and BAP-Zr (C_f_ = 1.20 mg/L).

Bioadsorbent		Fluoride Removal 1 h (%)	Fluoride Removal 24 h (%)	Arsenic Removal 24 h (%)
Well water	BOP-Zr	85	95	81
	BAP-Zr	69	93	84
Laboratory solution	BOP-Zr	64	86	82
	BAP-Zr	69	94	84

## Data Availability

Data are available only upon request to the authors.

## Supplementary Materials

The following supporting information can be downloaded at: https://www.mdpi.com/article/10.3390/polym14081575/s1, Figure S1: Bio adsorbent preparation process, Figure S2: Relationship between the Zr3d binding energy and the Pauling charge in the Zr species of Pantano and Brow [67] and of the present study; Table S1: Summary of parameters analyzed by the BET method, Table S2: Physico-chemical characterization of groundwater from the state of Durango, México, Table S3: Peak Deconvolution peaks on bioadsorbents before and after adsorption.
