# Supplementary material for: Zr-Based Biocomposite Materials as an Alternative for Fluoride Removal, Preparation and Characteristics
Source: Polymers (Basel). 2022 Apr 12;14(8):1575. doi: 10.3390/polym14081575 (PMC9025067; doi:10.3390/polym14081575)
Supplement: Supplementary file 1 [file polymers-14-01575-s001.zip › polymers-1634993-supplementary.pdf]

*Supplementary Materials*

# Zr-Based Biocomposite Materials as an Alternative for Fluoride Removal, Preparation and Characteristics

**Adriana Robledo-Peralta [1], Linda Viviana García-Quiñonez [2], René I. Rodríguez-Beltrán [2,*] and Liliana Reynoso-Cuevas [3,*]**

1 Department of Sustainable Engineering, Advanced Materials Research Center (CIMAV-Durango), CIMAV 110 street, Ejido Arroyo Seco, Durango C.P. 34147, Durango, Mexico; adriana.robledo@cimav.edu.mx
2 CONACYT-Centro de Investigación Científica y de Educación Superior de Ensenada, Unidad Foránea Monterrey, Alianza Centro 504, PIIT, Apodaca C.P. 66629, Nuevo León, Mexico; linda@cicese.mx
3 Catedras CONACYT, Advanced Materials Research Center (CIMAV-Durango), CIMAV 110 Street, Ejido Arroyo Seco, Durango C.P. 34147, Durango, Mexico
* Correspondence: rrodrigu@cicese.mx (R.I.R.-B.); liliana.reynoso@cimav.edu.mx (L.R.-C.)

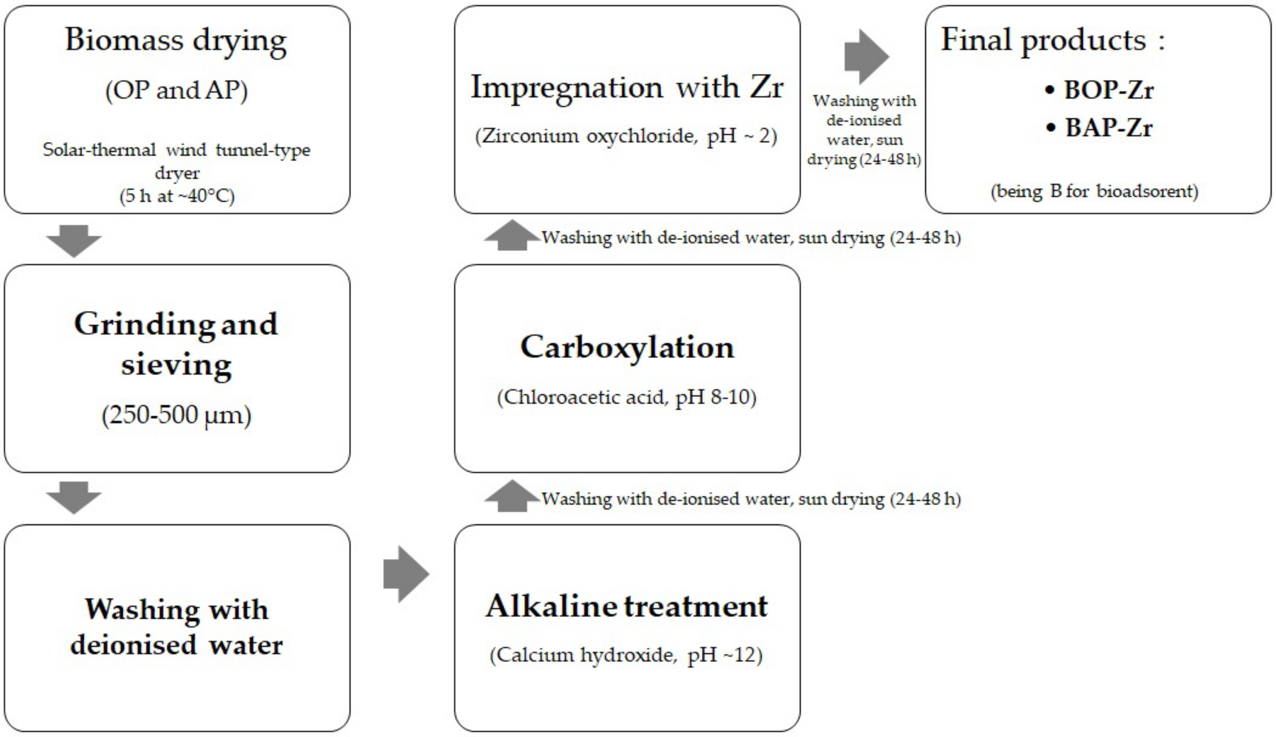


**Figure S1.** Bioadsorbent preparation process.

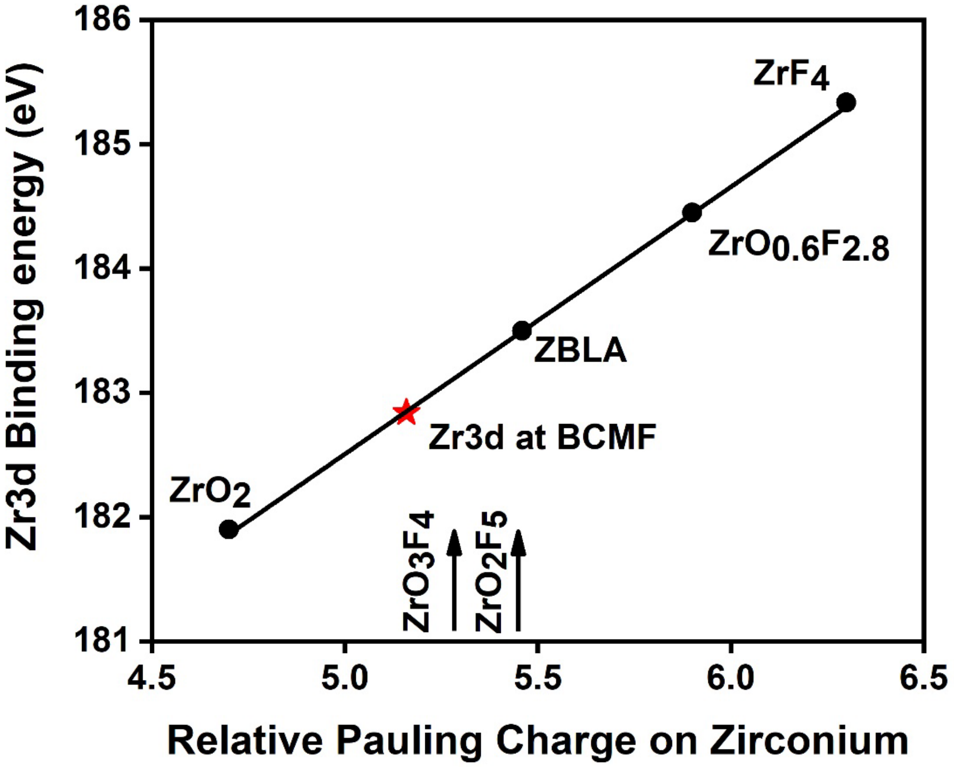


**Figure S2.** Relationship between the Zr3d binding energy and the Pauling charge in the Zr species of Pantano and Brow, 1988 [62] and of the present study ★.

**Table S1.** Summary of parameters analyzed by the BET method

| Sample | Surface area ($m^2/g$) | Pore volume ($cm^3/g$) | Pore size (nm) |
|---|---|---|---|
| BOP-Zr | 0.751 | 0.00141 | 6.070 |
| BAP-Zr | 0.521 | 0.00066 | 5.060 |

**Table S2.** Physico-chemical characterization of groundwater from the state of Durango, México.

| Parameter | Measured value |
|---|---|
| pH | 8.7 |
| Electrical conductivity (µs/cm) | 548.25 |
| DQO (mg $O_2$/L) | 104 |
| $Na^+$ (mg/L) | 53.10 |
| $K^+$ (mg/L) | 9.88 |
| $Ca^{+2}$ (mg/L) | 60.55 |
| $Mg^{+2}$ (mg/L) | 1.54 |
| $F^-$ (mg/L) | 3.89 |
| $NO_3^-$ (mg/L) | 38.36 |
| $NO_2^-$ (mg/L) | 1.84 |
| $Cl^-$ (mg/L) | 26.39 |
| $CO_3^{-2}$ (mg/L) | 0 |
| $HCO_3^-$ (mg $CaCO_3$/L) | 148.50 |
| $SO_4^{-2}$ (mg/L) | 59.76 |
| $As_{Total}$ (mg/L) | 0.032 |

$As_{Total}=As^{+5}+As^{+3}$

**Table S3.** Peak Deconvolution peaks on bioadsorbents before and after adsorption.

| Species | Peak´s binding energy (Ev) | | | | Area CPS.Ev | | | |
|---|---|---|---|---|---|---|---|---|
| | BOP-Zr | BOP-Zr-F | BAP-Zr | BAP-Zr-F | BOP-Zr | BOP-Zr-F | BAP-Zr | BAP-Zr-F |
| **C1s** (C-C, C-H) | 284.75 | 284.72 | 284.80 | 284.75 | 6738.19 | 7211.94 | 14292.11 | 9897.90 |
| **C1s** (C-O-C, C-OH) | 286.12 | 286.36 | 286.52 | 286.10 | 4183.31 | 6444.94 | 1430.80 | 3315.20 |
| **C1s** (C=O) | 287.70 | 288.11 | 288.80 | 288.37 | 2948.47 | 3419.43 | 309.94 | 675.61 |
| **O1s** ($O^{-2}$) | 530.30 | Disappear | 529.91 | 529.80 | 541.40 | - | 98.97 | 341.51 |
| **O1s** ($OH^{-}$) | 532.50 | 532.65 | 532.35 | 532.42 | 10747.82 | 13943.75 | 3452.99 | 6268.26 |
| **O1s** ($H_2O$) | 534.63 | Disappear | 534.18 | 534.40 | 858.92 | - | 145.58 | 159.04 |
| **O1s** (O-Fx) | - | 535.23 | - | - | - | 1191.43 | - | - |
| **$Zr3d_{5/2}$** | 182.84 | 182.87 | 182.63 | 182.84 | 1713.71 | 1646.20 | 404.64 | 952.61 |
| **$Zr3d_{5/2}$** | 184.85 | 184.79 | 184.85 | 185.08 | 1196.70 | 1030.79 | 215.57 | 474.95 |
| **$Zr3d_{3/2}$** | 185.53 | 185.54 | 185.00 | 185.41 | 521.89 | 709.44 | 148.36 | 150.17 |
| **$Zr3d_{3/2}$** | 186.94 | 187.04 | 186.73 | 185.35 | 242.20 | 523.78 | 47.05 | 103.35 |
| **$ZrF_4$** | - | 685.0 | - | 685.49 | - | 327.71 | - | 205.39 |
| **$ZrO_3F_4$** | - | 689.77 | - | 689.43 | - | 467.44 | - | 268.61 |
